# Ultrasound-guided percutaneous dilatational tracheostomy versus landmark- and bronchoscopy-guided techniques in critically ill patients: a systematic review and meta-analysis of randomized controlled trials

**DOI:** 10.3389/fmed.2026.1818411

**Published:** 2026-03-31

**Authors:** Binbin Ren, Yilu Bao, Honghang Lin, Bo Tang, Biyun Chen, Kai Zhang

**Affiliations:** 1 Department of Infectious Diseases, Affiliated Jinhua Hospital, Zhejiang University School of Medicine, Jinhua, China; 2 Department of Ultrasound Medicine, The First People's Hospital of Taizhou, Taizhou, China; 3 Department of Emergency Medicine, The First People's Hospital of Taizhou, Taizhou, China; 4 Department of Critical Care Medicine, Second Affiliated Hospital of Zhejiang University School of Medicine, Hangzhou, China

**Keywords:** bronchoscopy-guided, critically ill, landmark-guided, meta-analysis, percutaneous dilatational tracheostomy, systematic review, ultrasound-guided

## Abstract

**Background:**

Percutaneous dilatational tracheostomy (PDT) is widely performed in critically ill patients. In contemporary intensive care units (ICUs), multimodal image-guided PDT (particularly the combination of ultrasound and bronchoscopy) is increasingly advocated to maximize procedural safety. However, randomized data comparing single-modality guidance strategies remain clinically relevant, especially where one modality is unavailable.

**Methods:**

We conducted a systematic review and meta-analysis of randomized controlled trials (RCTs) comparing ultrasound-guided PDT with anatomical landmark-guided PDT or bronchoscopy-guided PDT. Four databases were searched from inception to February 16, 2026. Primary outcome was overall procedural complication rate. Secondary outcomes included first-attempt success rate, bleeding events, and procedure time. Random-effects models were used to pool risk ratios (RRs) or mean differences (MDs) with 95% confidence intervals (CIs).

**Results:**

A total of 11 RCTs (1,035 patients) were included. Compared with other percutaneous PDT techniques, ultrasound-guided PDT significantly reduced the risk of overall procedural complications (RR 0.56, 95% CI 0.46 to 0.69, *p* < 0.00001, I^2^ = 75%) improved the first-attempt success rate (RR 1.23, 95% CI 1.08 to 1.42, *p* = 0.003, I^2^ = 54%), and reduced the incidence of bleeding events (RR 0.44, 95% CI 0.29 to 0.67, *p* < 0.0001, I^2^ = 0%). No significant difference in procedure time was observed between ultrasound-guided and comparator techniques (MD −1.20, 95% CI −4.13 to 1.74, *p* = 0.42, I^2^ = 98%).

**Conclusion:**

In critically ill adults undergoing percutaneous tracheostomy, ultrasound guidance is associated with higher first-attempt success, lower risks of overall complications and bleeding, without prolonging procedure duration. These findings support ultrasound-guided PDT as a preferred percutaneous approach. Nevertheless, these findings should be interpreted within modern practice, where combined ultrasound plus bronchoscopy is often considered the preferred safety strategy in well-equipped ICUs, particularly for high-risk anatomy and re-intervention cases.

**Systematic review registration:**

https://osf.io/7wgzu, identifier.

## Introduction

Percutaneous dilatational tracheostomy (PDT) is a core bedside procedure in intensive care unit (ICU) for patients requiring prolonged mechanical ventilation or airway protection (1, 2). Compared with open surgical tracheostomy, PDT offers several logistical advantages in the ICU setting, including avoidance of patient transfer, reduced procedural invasiveness, and favorable peri-procedural outcomes in selected populations (3, 4). Nevertheless, PDT remains technically demanding and is associated with clinically significant complications, including bleeding, false passage, hypoxemia, and airway injury (5, 6). Because many of these adverse events are closely related to puncture accuracy and real-time anatomical visualization, the choice of guidance strategy has become a central determinant of procedural safety.

Three percutaneous guidance approaches are currently used in clinical practice: anatomical landmark-guided PDT, bronchoscopy-guided PDT, and ultrasound-guided PDT (7, 8). Landmark-guided PDT relies on external neck anatomy and operator experience; however, its accuracy may be compromised in patients with obesity, cervical edema, or distorted anatomy (9). Bronchoscopy-guided PDT enables intraluminal visualization and may help prevent posterior tracheal wall injury, but it introduces additional equipment burden and risks of airway resistance, hypercapnia, and procedural complexity (10, 11). By contrast, ultrasound-guided PDT permits pre-procedural and/or real-time visualization of cervical vessels, tracheal rings, the thyroid isthmus, and puncture trajectory, including skin-to-trachea depth measurement, potentially reducing vascular and puncture-related complications (12, 13).

Over the past decade, randomized and comparative studies have increasingly evaluated ultrasound-guided PDT versus other percutaneous techniques. Prior meta-analyses suggested that ultrasound guidance improves puncture accuracy and reduces peri-procedural complications, particularly bleeding, compared with landmark guidance (14–16). More recent evidence, including updated analyses and contemporary randomized controlled trials (RCTs) syntheses, continues to support a potential safety advantage of ultrasound guidance, while findings on procedure time remain inconsistent across studies (16, 17).

Importantly, contemporary ICU practice is evolving from “either/or” guidance toward multimodal safety strategies. In well-resourced units, combining preprocedural/real-time ultrasound with bronchoscopic intraluminal confirmation is increasingly promoted to reduce avoidable complications, including in technically challenging patients. Recent reports also suggest feasibility and safety of PDT under combined safety measures in high-risk settings, including patients with altered neck anatomy, prior neck surgery, or repeat tracheostomy (18, 19). However, RCT evidence remains largely structured as comparisons between single guidance modalities, and many ICUs worldwide still face constraints in equipment availability, staffing, or workflow implementation. Therefore, comparative effectiveness data between ultrasound, landmark, and bronchoscopy-guided PDT remain clinically relevant.

Given the increasing adoption of point-of-care ultrasound and the emergence of newer randomized data, we conducted an updated RCT-focused meta-analysis comparing ultrasound-guided PDT with landmark-guided and bronchoscopy-guided PDT in critically ill adults, focusing on first-attempt success, overall complications, bleeding, and procedure time.

## Methods

### Protocol registration and reporting standards

This systematic review and meta-analysis was conducted in accordance with the Preferred Reporting Items for Systematic Reviews and Meta-Analyses (PRISMA) 2020 statement (20) (see Supplementary material 1 for the checklist). The study protocol was prospectively registered on the Open Science Framework platform.^1^

### Data sources and search strategy

A comprehensive literature search was performed across four electronic databases (PubMed, Embase, Scopus, and Cochrane Library) from inception to February 16, 2026. Search terms combined controlled vocabulary and free-text keywords related to tracheostomy and guidance modalities, including: “tracheostomy,” “ultrasonography,” “ultrasound-guided,” and “randomized controlled trial”. The complete search strategy for each database is provided in Supplementary material 2. In addition, references of eligible studies and relevant reviews were manually screened to identify additional RCTs.

### Study eligibility criteria

Studies were included if they met all of the following criteria:

*Population*: Adult critically ill patients undergoing PDT, predominantly in intensive care settings.

*Intervention*: Ultrasound-guided PDT, including real-time and/or preprocedural ultrasound-assisted localization.

*Comparator*: Other percutaneous techniques, including anatomical landmark-guided PDT and bronchoscopy-guided PDT.

*Outcomes*: Primary outcomes included and overall peri-procedural complication rate. Secondary outcomes included first-attempt tracheostomy success rate, bleeding events and procedure time.

*Study design*: RCTs published in English.

Studies were excluded if they were non-randomized, enrolled pediatric populations, compared surgical tracheostomy only, contained duplicate datasets, or lacked extractable outcome data. Case reports, narrative reviews, and editorials were also excluded.

### Study selection and data extraction

**Figure 1:**
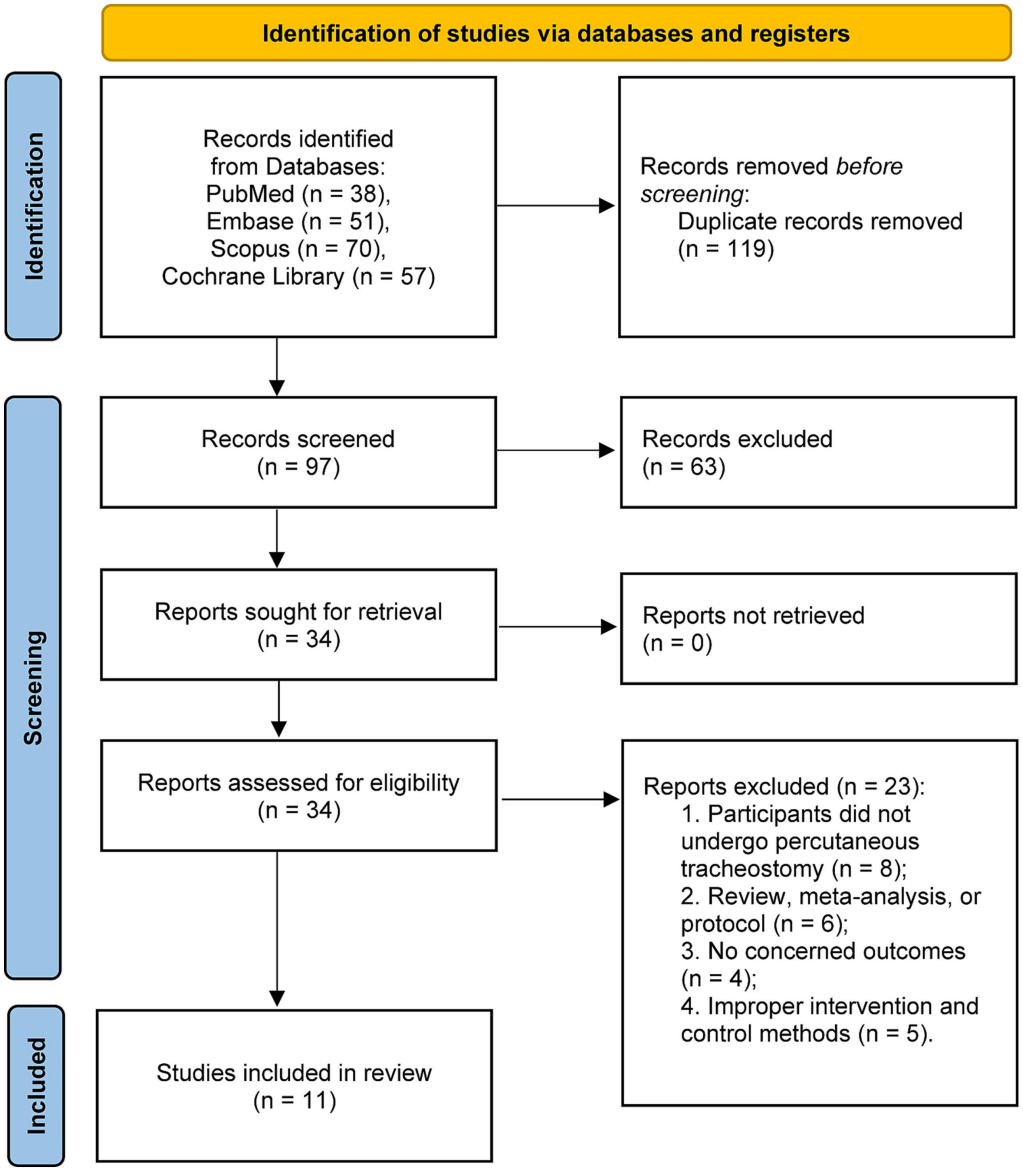
PRISMA 2020 flow diagram for this systematic review and meta-analysis.

Two reviewers (Yilu Bao, Honghang Lin) independently screened records in two sequential stages: (1) title and abstract screening and (2) full-text eligibility assessment. Disagreements were resolved by discussion; a third reviewer adjudicated any unresolved conflicts. The selection process was documented using a PRISMA flow diagram (Figure 1).

Data were extracted independently by two reviewers (Yilu Bao, Bo Tang) using a pre-designed standardized form. Extracted items included study characteristics (first author, year, country), sample size, and patient characteristics. Where data were incomplete or reported in non-standard formats, required statistics were derived from published information or obtained by contacting corresponding authors where feasible.

### Risk of bias assessment

The risk of bias was independently evaluated by two reviewers (Yilu Bao, Honghang Lin) utilizing the Cochrane Risk of Bias 2.0 tool (21). This assessment covered five domains: the randomization process, deviations from intended interventions, missing outcome data, outcome measurement, and selection of reported results. For each domain and subsequently for each study as a whole, a judgment of “low risk,” “some concerns,” or “high risk” of bias was determined. Any disagreements between the reviewers were settled through consensus.

### Data synthesis and statistical analysis

To address anticipated clinical and methodological heterogeneity, a random-effects model was employed for the meta-analysis. For dichotomous outcomes, pooled risk ratios (RRs) with corresponding 95% confidence intervals (CIs) were computed; for continuous outcomes, pooled mean differences (MDs) with 95% CIs were calculated. The extent of statistical heterogeneity was evaluated using the Cochran Q test and quantified via the I^2^ statistic, where values of 25, 50, and 75% were predefined as thresholds for low, moderate, and substantial heterogeneity, respectively (22).

Where sufficient data were available, prespecified subgroup analyses were undertaken based on comparator type (anatomical landmark-guided versus bronchoscopy-guided). To assess the robustness of the pooled estimates, sensitivity analyses were conducted by iteratively removing individual studies and by excluding trials judged to be at a higher risk of bias.

The potential for publication bias was assessed using Egger’s weighted regression test and through visual examination of funnel plot symmetry (23). In instances where funnel plot asymmetry indicated possible small-study effects, a trim-and-fill adjustment was applied to examine the stability of the summary estimates under different assumptions regarding bias (24). All statistical analyses were executed using RevMan software and the R statistical environment (specifically the “meta” and “robvis” packages) (25, 26). A two-sided *p* value <0.05 was defined as statistically significant.

## Results

### Study selection and study characteristics

The study selection process is detailed in the PRISMA flow diagram (Figure 1). A total of 216 records were initially identified through systematic searches of four databases: PubMed (*n* = 38), Embase (*n* = 51), Scopus (*n* = 70), and the Cochrane Library (*n* = 57). After removal of 119 duplicates, 97 unique records were screened by title and abstract. Of these, 63 were excluded, leaving 34 reports retrieved for full-text assessment. Following full-text review, 23 articles were excluded for various reasons, and 11 studies met all pre-specified eligibility criteria and were included in the final analysis (17, 27–36).

The 11 RCTs comprised five studies comparing ultrasound-guided PDT with anatomical landmark-guided PDT, and six comparing ultrasound-guided PDT with bronchoscopy-guided PDT. In aggregate, 1,035 patients were enrolled: 524 in the ultrasound-guided PDT group, 300 in the anatomical landmark-guided PDT group, and 211 in the bronchoscopy-guided PDT group. Baseline characteristics of the included studies are summarized in Table 1. All enrolled participants were critically ill adults receiving mechanical ventilation in an ICU or high-dependency unit with an indication for PDT. Studies were conducted across seven countries (Australia, Turkey, India, Brazil, Egypt, and Pakistan) and published between 2014 and 2025.

**Table 1:** Characteristics of included studies.

Study, year, and country	Technique and number	Population
Rudas, 2014 (33), Australia	Ultrasound-guided: 25; Anatomical landmark-guided: 25	Adult intensive care patients received mechanical ventilation, required tracheostomy
Yavuz 2014 (36), Turkey	Ultrasound-guided: 166; Anatomical landmark-guided: 175	Adult intubated critically ill patients in ICU, received mechanical ventilation, required tracheostomy
Ravi, 2015 (32), India	Ultrasound-guided: 38; Bronchoscopy-guided: 36	Adult patients in ICU or HDU, received mechanical ventilation, required tracheostomy
Gobatto, 2016 (28), Brazil	Ultrasound-guided: 60; Bronchoscopy-guided: 58	Adult intubated critically ill patients received mechanical ventilation, indicated for tracheostomy
Kupeli 2017 (30), Turkey	Ultrasound-guided: 40; Anatomical landmark-guided: 20	Adult critically ill patients on prolonged mechanical ventilation in ICU
Sarıtaş 2019 (34), Turkey	Ultrasound-guided: 40; Bronchoscopy-guided: 40	Adult critically ill patients dependent on mechanical ventilation in ICU
Elazzazi, 2020 (27), Egypt	Ultrasound-guided: 20; Bronchoscopy-guided: 20	Adult patients required tracheostomy in critical care department
Dugg, 2022 (17), India	Ultrasound-guided: 50; Anatomical landmark-guided: 50	Adult critically ill patients on prolonged mechanical ventilation
Kumar, 2022 (29), India	Ultrasound-guided: 30; Anatomical landmark-guided: 30	Adult critically ill patients needed tracheostomy after prolonged ventilator support
Nazir, 2022 (31), Pakistan	Ultrasound-guided: 25; Bronchoscopy-guided: 27	Adult critically ill patients on prolonged mechanical ventilation in ICU
Taha, 2025 (35), Egypt	Ultrasound-guided: 30; Bronchoscopy-guided: 30	Adult intubated and mechanically ventilated critically ill patients admitted to the ICU

ICU, intensive care unit; HDU, high dependency unit.

### Risk of bias assessment

**Figure 2:**
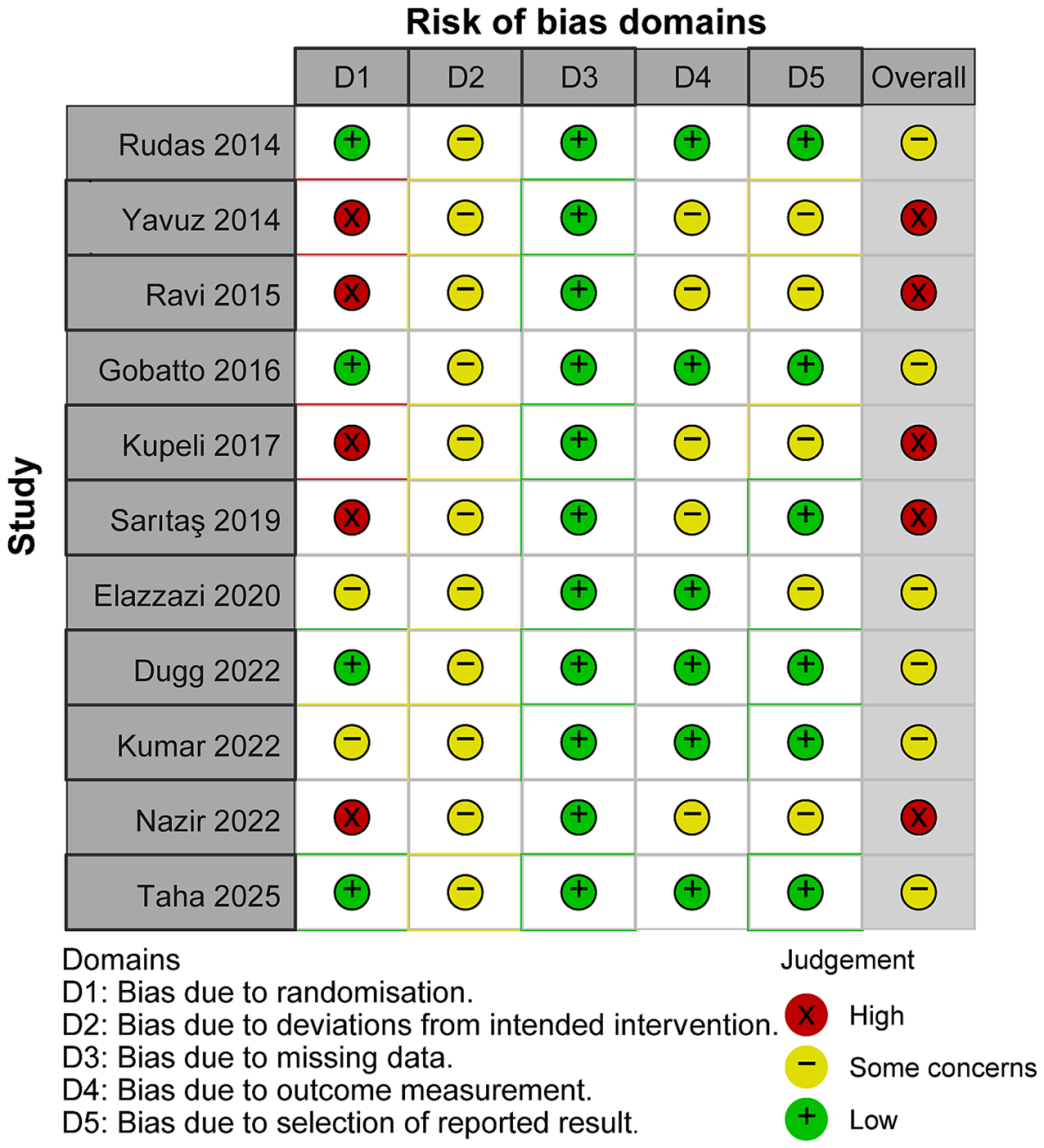
Risk of bias 2 of all included studies.

Risk of bias was assessed for all 11 included RCTs using the Cochrane Risk of Bias 2 tool (Figure 2). Overall, 6 trials were rated as “some concerns” and 5 as “high risk.”

At the domain level, the most prominent limitations were identified in Domain 1 (randomization process) and Domain 2 (deviations from intended interventions). In Domain 1, several studies were rated high risk owing to insufficient reporting or potential imbalance in allocation procedures. In Domain 2, all studies were rated as having some concerns, primarily reflecting the inherent difficulty of blinding in PDT trials. Domain 3 (missing outcome data) was generally robust, with all studies rated low risk. For Domains 4 (outcome measurement) and 5 (selection of reported results), most studies were rated low risk, with the remainder having some concerns. Overall, methodological limitations related to randomization and trial conduct are the primary constraints on the certainty of pooled estimates.

Publication bias was formally assessed for all four outcomes using funnel plot inspection and Egger’s test (Supplementary material 3). For first-attempt success rate, funnel plot asymmetry was visually apparent; trim-and-fill analysis yielded an adjusted pooled estimate of RR 1.13 (95% CI 0.98 to 1.30), which was no longer statistically significant, suggesting that the original pooled effect may have been inflated by selective publication. Similarly, Egger’s test identified significant asymmetry for overall complication rate (*p* = 0.034); trim-and-fill analysis produced an adjusted estimate of RR 0.77 (95% CI 0.50 to 1.20), which also failed to reach significance after imputation of putatively missing studies. In contrast, neither funnel plot inspection nor Egger’s test indicated significant publication bias for bleeding risk or procedure time, and pooled estimates for these outcomes were considered more robust. These findings indicate that results pertaining to first-attempt success rate and overall complication rate should be interpreted with caution, whereas estimates for bleeding risk and procedure time are less susceptible to publication bias.

### Outcomes

#### Overall procedural complications

**Figure 3:**
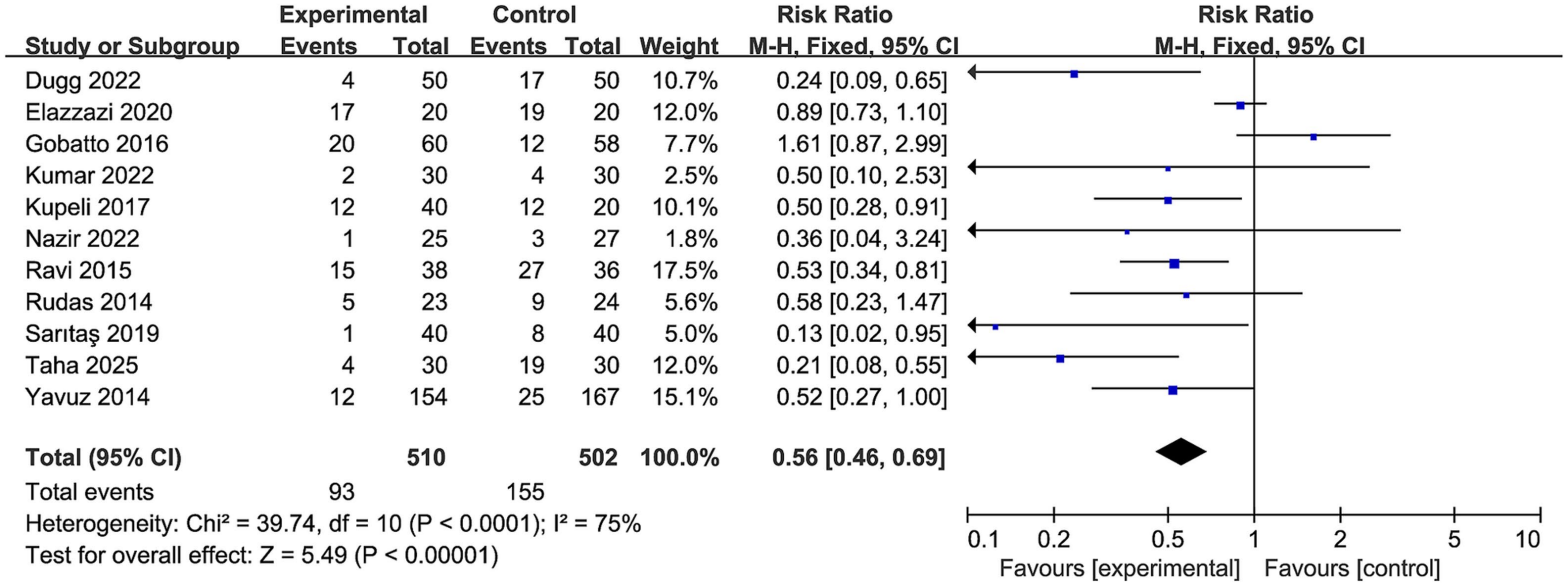
Forest plot comparing the effect of ultrasound-guided PDT versus anatomical landmark-guided PDT and/or bronchoscopy-guided PDT on overall procedural complication rate.

All 11 RCTs reported overall peri-procedural complication rates, encompassing events such as minor and major bleeding, transient oxygen desaturation, transient hypotension, endotracheal tube cuff puncture, subcutaneous emphysema, and pneumothorax, as defined by individual study authors. Ultrasound-guided PDT was associated with a statistically significant 44% relative reduction in overall complications compared with non-ultrasound-guided approaches (RR 0.56, 95% CI 0.46 to 0.69, *p* < 0.00001, I^2^ = 75%, Figure 3).

#### First-attempt tracheostomy success

**Figure 4:**
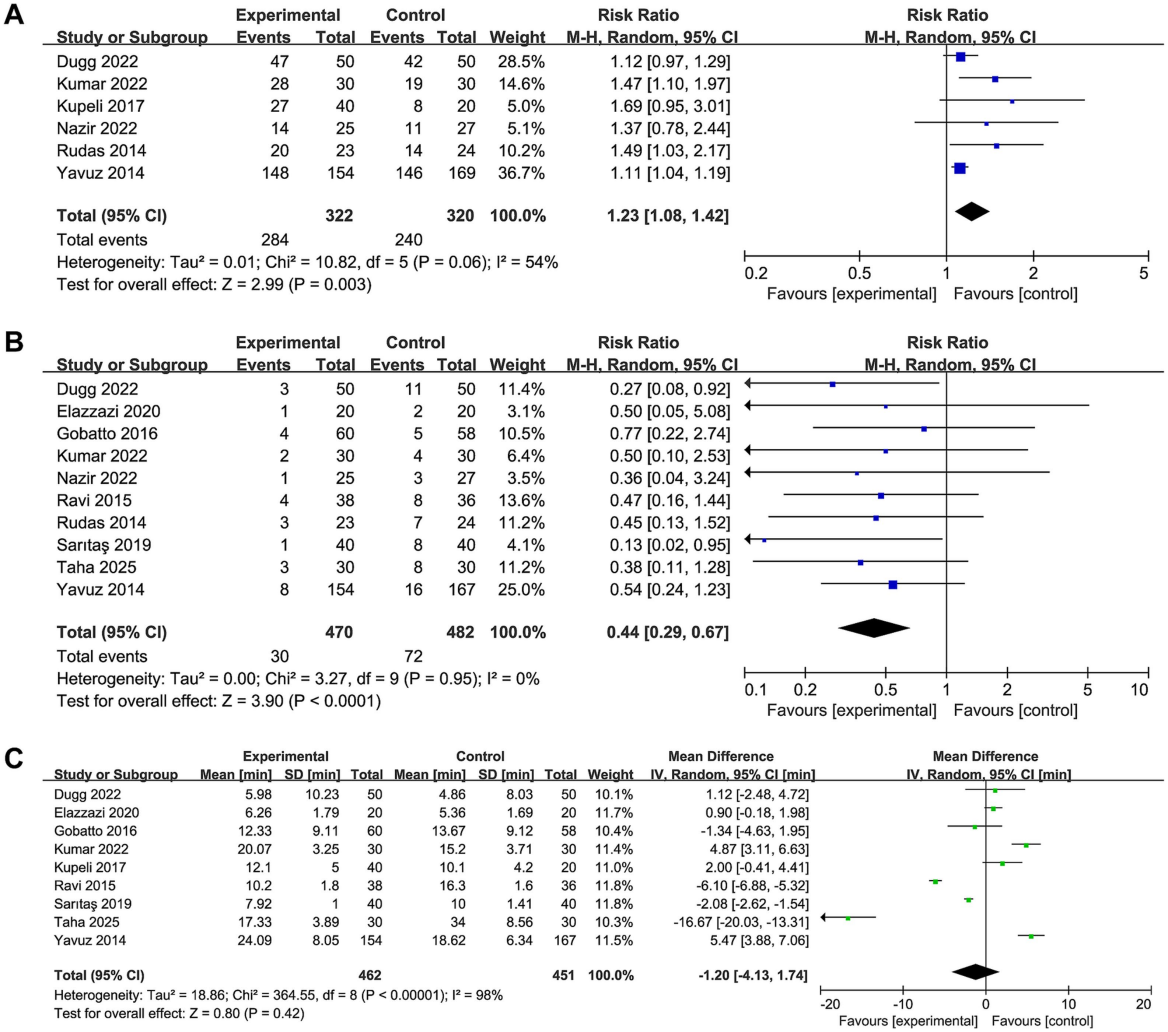
Forest plot comparing the effect of ultrasound-guided PDT versus anatomical landmark-guided PDT and/or bronchoscopy-guided PDT on **(A)** first-attempt success rate, **(B)** bleeding events and **(C)** procedure time.

Seven of 11 studies reported first-attempt success rate data. Ultrasound-guided PDT was associated with a significantly higher first-puncture success rate compared with non-ultrasound-guided approaches (RR 1.23, 95% CI 1.08 to 1.42, *p* = 0.003, I^2^ = 54%, Figure 4A), representing approximately a 23% greater likelihood of successful tracheal cannulation on the first puncture attempt with ultrasound guidance.

#### Bleeding events

Nine of 11 studies reported procedural bleeding data, encompassing both minor bleeding (controlled with local compression, not requiring transfusion or surgical intervention) and major bleeding (hemodynamically significant hemorrhage requiring transfusion, surgical revision, or resulting in hemodynamic instability). Ultrasound-guided PDT was associated with a significantly lower risk of bleeding compared with comparator techniques (RR 0.44, 95% CI 0.29 to 0.67, *p* < 0.0001, I^2^ = 0%, Figure 4B), representing a 56% relative reduction in bleeding risk.

#### Procedure time

Ten of 11 studies reported procedure time, which was variably defined as the interval from skin incision to tube placement, from first needle puncture to tube confirmation, or from initiation of guided assessment to procedural completion. No statistically significant difference in procedure time was identified between between ultrasound-guided and non-ultrasound-guided techniques (MD −1.20, 95% CI −4.13 to 1.74, *p* = 0.42, I^2^ = 98%, Figure 4C). The substantial heterogeneity for this outcome is attributable to the absence of a standardized definition for procedural timing, as well as differences in operator experience, institutional protocols, and whether pre-procedural ultrasonographic scanning time was included in the reported duration.

### Sensitivity and subgroup analyses

Leave-one-out sensitivity analyses were performed for all four outcomes by sequentially omitting each contributing study (Supplementary material 3). For first-attempt success rate, recalculated pooled RRs across all iterations ranged from 1.13 to 1.32, with all estimates retaining statistical significance (*p* < 0.01), confirming stability of the primary finding. For overall complication rate, pooled RRs across leave-one-out models ranged from 0.49 to 0.60, with all iterations maintaining statistical significance (*p* < 0.01) and a consistently protective direction. For bleeding risk, pooled estimates were highly stable, with recalculated RRs ranging from 0.41 to 0.47 (*p* < 0.01 for all iterations), reflecting negligible between-study variability. For procedure time, no leave-one-out iteration yielded a statistically significant pooled MD (*p* = 0.36 to 0.79), confirming that the null finding was not attributable to any single trial.

Pre-specified subgroup analyses stratified by comparator technique yielded clinically informative patterns (Supplementary material 3). For first-attempt success rate, ultrasound-guided PDT was significantly superior to anatomical landmark-guided PDT, whereas no significant difference was observed versus bronchoscopy-guided PDT, indicating that the advantage of ultrasound guidance in cannulation accuracy is most evident relative to unguided approaches. A consistent pattern was observed for overall complication rate: ultrasound-guided PDT significantly reduced complication rates compared with anatomical landmark-guided PDT but not bronchoscopy-guided PDT, suggesting that the safety advantage of ultrasound-guided PDT is primarily driven by its superiority over techniques lacking real-time anatomical visualization. In contrast, the reduction in bleeding risk with ultrasound-guided PDT was statistically significant in both subgroups, indicating that ultrasound’s capacity to identify and avoid anterior cervical vasculature confers a hemostatic advantage not replicated by bronchoscopic guidance. For procedure time, the effects of ultrasound-guided PDT were directionally opposing across subgroups: ultrasound-guided PDT required significantly more time than anatomical landmark-guided PDT, reflecting the additional time for ultrasonographic assessment, but significantly less time than bronchoscopy-guided PDT, likely owing to the greater logistical complexity of bronchoscopic setup. Collectively, these subgroup findings demonstrate that the comparative effectiveness and safety profile of ultrasound-guided PDT are substantially modulated by the nature of the comparator technique.

## Discussion

### Principal findings

This systematic review and meta-analysis synthesized RCT evidence comparing ultrasound-guided PDT with anatomical landmark-guided PDT and bronchoscopy-guided PDT. The principal findings are as follows. First, ultrasound-guided PDT significantly reduced overall peri-procedural complication rates compared with anatomical landmark-guided PDT but not bronchoscopy-guided PDT. Second, ultrasound-guided PDT was associated with a significantly higher first-attempt success rate compared with anatomical landmark-guided PDT, with no statistically significant difference observed relative to bronchoscopy-guided PDT, indicating that real-time ultrasound guidance meaningfully improves procedural accuracy over unguided approaches while achieving cannulation precision comparable to bronchoscopic visualization. Third, ultrasound-guided PDT demonstrated a consistent and statistically significant reduction in bleeding risk relative to both anatomical landmark-guided PDT and bronchoscopy-guided PDT, suggesting that ultrasound’s extraluminal vascular mapping capability confers a hemostatic advantage not replicated by either comparator modality. Fourth, the effect of ultrasound-guided PDT on procedure time was heterogeneous and directionally opposing across subgroups, with ultrasound-guided PDT requiring more time than anatomical landmark-guided PDT but less than bronchoscopy-guided PDT, yielding a non-significant primary pooled estimate. Sensitivity analyses confirmed the robustness of these findings. Collectively, these results support the value of real-time ultrasound guidance as a safe and effective approach for PDT, with particular benefit in hemorrhagic complication prevention.

### Comparison with existing literature

The present findings are broadly consistent with and extend the existing literature on PDT. Prior systematic reviews and meta-analyses have reported that ultrasound guidance improves procedural accuracy and reduces complication rates relative to landmark-based techniques (7, 8, 16). However, the available evidence has been characterized by considerable methodological heterogeneity, inconsistent outcome reporting, and a failure to distinguish between different comparator guidance modalities, limitations that constrain the interpretability of previously published pooled estimates.

The observed superiority of ultrasound-guided PDT over anatomical landmark-guided PDT in first-attempt success rate is consistent with the fundamental rationale for real-time imaging in tracheostomy practice. Anatomical landmark-guided PDT relies on surface palpation to estimate underlying tracheal anatomy, a technique inherently susceptible to error in patients with obesity, prior cervical surgery, short neck morphology, or abnormal tracheal positioning (37). Ultrasound circumvents these limitations by enabling direct pre-procedural visualization of tracheal cartilaginous rings, the cricothyroid membrane, the thyroid gland, and peritracheal soft tissues, thereby facilitating accurate midline identification and optimal needle trajectory planning (38). The equivalence of ultrasound-guided PDT and bronchoscopy-guided PDT in first-attempt success rate is biologically plausible: bronchoscopic guidance provides real-time intraluminal tracheal visualization, enabling confirmation of needle placement under direct endoscopic view and thus achieving comparable procedural accuracy through a fundamentally different mechanism (7).

The finding that ultrasound-guided PDT significantly reduces bleeding risk compared with both anatomical landmark-guided PDT and bronchoscopy-guided PDT is a particularly important contribution of the present analysis. Although individual studies have reported reduced vascular injury rates with ultrasound guidance (17, 39), no previous meta-analysis has systematically demonstrated this advantage over bronchoscopic guidance in a pooled analysis stratified by comparator technique. The mechanistic basis is compelling: ultrasonography enables pre-procedural color Doppler mapping of aberrant or overlying anterior jugular veins, thyroid vessels, and other cervical vascular structures that are not discernible by external palpation and are invisible to the bronchoscopist operating within the tracheal lumen (40). These findings provide mechanistic and empirical support for preferential use of ultrasound guidance in patients with anticipated cervical vascular anomalies, coagulopathy, or elevated hemorrhagic risk (41, 42).

The heterogeneous effect of ultrasound-guided PDT on procedure time is consistent with prior literature. The additional time required for ultrasonographic scanning and real-time image interpretation necessarily prolongs the procedure compared with the more expedient landmark-guided approach (43). Conversely, the portability and single-operator capability of ultrasound confer a time advantage over bronchoscopy-guided PDT, which requires dedicated bronchoscope preparation and bronchoscopist attendance (44).

### Clinical implications

Our findings should be interpreted in the context of current ICU standards. First, the superiority of ultrasound-guided PDT over landmark-guided PDT in first-attempt success and complication reduction supports abandoning unguided puncture whenever imaging is available. Second, although ultrasound-guided PDT showed comparable major performance outcomes to bronchoscopy-guided PDT in this RCT synthesis, this does not imply that bronchoscopy is unnecessary in modern ICUs. On the contrary, in well-equipped settings, accumulating practice-based evidence supports a combined ultrasound + bronchoscopy strategy to maximize safety, particularly in patients with obesity, coagulopathy, distorted neck anatomy, prior neck surgery, or repeat tracheostomy (18, 19). Third, the practical “choice” between ultrasound-only and bronchoscopy-only guidance is most relevant in settings where one modality is not available. In such scenarios, our pooled results provide comparative evidence for decision-making. Where both modalities are available, a multimodal approach should be considered the preferred framework rather than strict modality substitution.

Second, the finding that ultrasound-guided PDT achieves comparable first-attempt success and overall complication rates to bronchoscopy-guided PDT, while conferring a significant additional advantage in bleeding prevention. Bronchoscopy-guided tracheostomy has historically been considered the gold standard for image-guided airway management; however, it is associated with increased procedural cost, the requirement for a dedicated bronchoscopist, risks of transient hypoxemia and hypercarbia, and resource constraints in some institutions (45). The present findings suggest that ultrasound-guided PDT may represent a clinically equivalent or superior alternative to bronchoscopy-guided PDT in most clinical scenarios, particularly in patients at elevated hemorrhagic risk, while offering logistical and resource advantages. For high-risk patients including those with obesity, coagulopathy, prior neck surgery, distorted cervical anatomy, or known vascular anomalies, ultrasound guidance should be considered the preferred primary modality, with bronchoscopy reserved as an adjunctive or confirmatory tool where clinically indicated (46).

## Limitations

Several limitations of this systematic review and meta-analysis warrant consideration. First, considerable heterogeneity was identified across included studies in terms of study design, patient population characteristics, operator experience, tracheostomy kit and technique, and outcome definitions. Although random-effects models and subgroup and sensitivity analyses were employed to explore and account for between-study variability, residual unexplained heterogeneity persisted for several outcomes, most prominently for overall complication rate and procedure time. Second, the included studies varied substantially in sample size, follow-up duration, and the specific definitions of key outcomes, including the threshold for classifying a complication, the method of bleeding assessment, and the measurement time point for procedure duration, thereby limiting comparability across studies and introducing potential outcome ascertainment bias. Third, formal assessment identified potential publication bias for first-attempt success rate and overall complication rate, with trim-and-fill analyses yielding attenuated and non-significant adjusted estimates for both outcomes; the pooled estimates for these endpoints should therefore be interpreted with caution. Fourth, the present analysis was restricted to studies reporting quantitative outcome data amenable to meta-analytic pooling; studies with insufficient or incompatible data reporting were excluded, which may have introduced a degree of selective inclusion bias. Finally, the majority of included studies were conducted in high-income settings with experienced operators, and the generalizability of these findings to resource-limited environments, lower-volume centers, or operators earlier in their training curve may be restricted. An additional limitation is that nearly all included RCTs compared single guidance modalities (ultrasound vs. landmark or bronchoscopy), whereas current high-resource ICU practice increasingly favors combined guidance techniques. Therefore, our pooled estimates cannot directly quantify the incremental benefit of ultrasound-plus-bronchoscopy protocols over either modality alone.

## Conclusion

This RCT-based meta-analysis shows that ultrasound-guided PDT improves first-attempt success and reduces complications and bleeding compared with landmark-guided PDT, and offers comparable overall performance to bronchoscopy-guided PDT with a potential bleeding advantage. These data support ultrasound as a strong core guidance modality. However, in modern well-equipped ICUs, the safest procedural paradigm is increasingly a multimodal approach integrating ultrasound and bronchoscopy, especially for high-risk or anatomically complex patients. Future randomized studies should directly compare combined versus single-modality guidance strategies.

## Data Availability

The original contributions presented in the study are included in the article/Supplementary material, further inquiries can be directed to the corresponding author.

## Footnotes

1
https://osf.io/7wgzu
